# The Role of Adenosine Receptors in Psychostimulant Addiction

**DOI:** 10.3389/fphar.2017.00985

**Published:** 2018-01-10

**Authors:** Inmaculada Ballesteros-Yáñez, Carlos A. Castillo, Stefania Merighi, Stefania Gessi

**Affiliations:** ^1^Department of Inorganic and Organic Chemistry and Biochemistry, School of Medicine, University of Castilla-La Mancha, Ciudad Real, Spain; ^2^Department of Nursing, Physiotherapy and Occupational Therapy, School of Nursing and Physiotherapy, University of Castilla-La Mancha, Toledo, Spain; ^3^Department of Medical Sciences, Pharmacology Section, University of Ferrara, Ferrara, Italy

**Keywords:** Adenosine A_1_ receptors, Adenosine A_2A_ receptors, amphetamines, cocaine, dopamine, psychostimulants, behavioral effects, striatum

## Abstract

Adenosine receptors (AR) are a family of G-protein coupled receptors, comprised of four members, named A_1_, A_2A_, A_2B_, and A_3_ receptors, found widely distributed in almost all human body tissues and organs. To date, they are known to participate in a large variety of physiopathological responses, which include vasodilation, pain, and inflammation. In particular, in the central nervous system (CNS), adenosine acts as a neuromodulator, exerting different functions depending on the type of AR and consequent cellular signaling involved. In terms of molecular pathways and second messengers involved, A_1_ and A_3_ receptors inhibit adenylyl cyclase (AC), through G_i/o_ proteins, while A_2A_ and A_2B_ receptors stimulate it through G_s_ proteins. In the CNS, A_1_ receptors are widely distributed in the cortex, hippocampus, and cerebellum, A_2A_ receptors are localized mainly in the striatum and olfactory bulb, while A_2B_ and A_3_ receptors are found at low levels of expression. In addition, AR are able to form heteromers, both among themselves (e.g., A_1_/A_2A_), as well as with other subtypes (e.g., A_2A_/D_2_), opening a whole range of possibilities in the field of the pharmacology of AR. Nowadays, we know that adenosine, by acting on adenosine A_1_ and A_2A_ receptors, is known to antagonistically modulate dopaminergic neurotransmission and therefore reward systems, being A_1_ receptors colocalized in heteromeric complexes with D_1_ receptors, and A_2A_ receptors with D_2_ receptors. This review documents the present state of knowledge of the contribution of AR, particularly A_1_ and A_2A_, to psychostimulants-mediated effects, including locomotor activity, discrimination, seeking and reward, and discuss their therapeutic relevance to psychostimulant addiction. Studies presented in this review reinforce the potential of A_1_ agonists as an effective strategy to counteract psychostimulant-induced effects. Furthermore, different experimental data support the hypothesis that A_2A_/D_2_ heterodimers are partly responsible for the psychomotor and reinforcing effects of psychostimulant drugs, such as cocaine and amphetamine, and the stimulation of A_2A_ receptor is proposed as a potential therapeutic target for the treatment of drug addiction. The overall analysis of presented data provide evidence that excitatory modulation of A_1_ and A_2A_ receptors constitute promising tools to counteract psychostimulants addiction.

## Introduction

Drug addiction is a complex chronic cognitive disorder characterized by drug seeking and compulsive use, which is difficult to control despite its harmful consequences. According to DSM-5 (American Psychiatric Association, 2013), which is used to define mental disorders in epidemiologic studies, it is now accepted that the criteria used to clinically define the terms *abuse* and *dependence* should be combined to form a new category known as *Substance use disorders*, including *craving* as a new criterion to increase diagnostic accuracy (Hasin et al., 2013). In terms of epidemiology, drug addiction is currently a global health problem, as can be deduced from comparison of data obtained from the Global Burden of Diseases Study between 1990 and 2015. For the period 1990–2015, global exposure to drug use increased by 30.2% for both sexes. In addition, by 2015 drug use was a major risk factor for early death and disability in developed countries like the United States, Canada, Australia, and the United Kingdom, being the 5th leading global risk factor for men and the 12th for women (GBD 2015 Risk Factors Collaborators, 2016).

Psychostimulants are a broad class of drugs whose effects include increases in arousal, wakefulness, cardiovascular stimulation, vigilance, and attention, and which constitute one of the most abused classes of prohibited drugs in the world, including as representative examples cocaine and amphetamine-like molecules (Chesworth et al., 2016). According to the 2017 report of the European Monitoring Centre for Drugs and Drug Addiction (European Monitoring Centre for Drugs and Drug Addiction, 2017), it was estimated that in the year 2016, the global annual prevalence among Europeans aged 15 or over was 3.5 million users of cocaine, 2.7 million users of MDMA and 1.8 million users of amphetamines which corresponds to 1.0, 0.8, and 0.5% of the European adults, respectively, which occasional consumed mentioned psychostimulants during 2016.

To date, the therapies developed to manage drug addiction are inadequate and unsatisfactory, and many scientists around the world are focusing on new strategies to improve them. Even though numerous aspects of this phenomenon are not well understood, the neurochemical mechanism common to all drugs causing abuse in humans is the increase of the neurotransmitter dopamine (DA) released from the ventral tegmental area (VTA), to a region in the mesocorticolimbic part of the brain, like the nucleus accumbens (NAc) and the prefrontal cortex (Filip et al., 2012; Morales and Margolis, 2017). This, in turn, increases the physiological reward and reinforcement mechanisms (Nestler and Landsman, 2001). This point is particularly important because DA not only mediates the effects of acute rewarding, but is also thought to be involved in the increased motivation to consume psychostimulants in psychostimulant abusers (Volkow et al., 2012). Furthermore, abuse of psychostimulants may induce changes in brain regions not only with relevance for addictive behavior, but may also promote long-term adverse consequences in areas related to memory and cognition (Nyberg, 2014). In addition, relapse into drug use after abstinence has been attributed to exposure to cues, stress or re-exposure to the drug itself that induce drug craving; the incubation of craving being a common phenomenon reported for most drugs of abuse, including psychostimulants, that may last from the beginning of abstinence for extended periods of time. Although little is known about the molecular mechanisms that lead to the incubation of craving during drug abstinence, vulnerability to relapse correlates with changes in the activity and structure of neurons from the limbic and frontal cortical circuitry, induced by the drug use (Pickens et al., 2011; Wolf, 2016).

The repeated ingestion of psychostimulants, as for most substances with marked abuse potential, shares one of the following two common features consistently reported in the literature: on the one hand, psychostimulants, by blocking molecular reuptake, enhance the extracellular neurotransmitter concentration in the synapses of monoaminergic neurons (Cooper et al., 1996; Rothman and Baumann, 2003; Wood et al., 2014); on the other hand, psychostimulants increase DA release in the NAc, a critical area for the reward circuit (Preedy, 2016). There is a growing body of scientific evidence demonstrating that psychostimulants affect dopaminergic neurons in the limbic reward system, and that this effect underlies addiction to stimulants (Siciliano et al., 2015).

Adenosine, an ubiquitous endogenous nucleoside, has been implicated in the reward-related behavior, and represents a novel and interesting target to interfere with it, as a consequence of its modulatory function on neurotransmission exerted by DA, glutamate and acetylcholine (Linden, 2001; Cunha, 2005; Gomes et al., 2011; Lopes et al., 2011; Borea et al., 2016; Burnstock, 2017; Jacobson et al., 2017). Interestingly, adenosine levels are modified following acute or chronic consumption of drugs of abuse and psychostimulants (Hack and Christie, 2003; Brown and Short, 2008; Filip et al., 2012), suggesting that a better comprehension of adenosine signaling in the brain during addiction may open new pharmacological frontiers to explore potential treatments in preclinical studies and clinical trials over the next years (Stone, 1981; Clark and Dar, 1989; Krauss et al., 1993; Bonci and Williams, 1996; Salem and Hope, 1999). The question of whether adenosine signaling can be used as a potential therapy in abuse disorders remains to be answered. Therefore, the goal of this review is to discuss current scientific evidence based on animal models of psychostimulant addiction, and to suggest promising candidates in the search for pharmacological interventions. We will include, when available, the effects of adenosine receptor (AR) ligands on the complex process of behavior related to psychostimulant consumption, seeking, withdrawal, craving and relapse, and we will restrict our discussion to what we can consider psychostimulant drugs, namely cocaine and amphetamine-like molecules.

## Brain Circuitry and Adenosine Receptor Interactions

**FIGURE 1:**
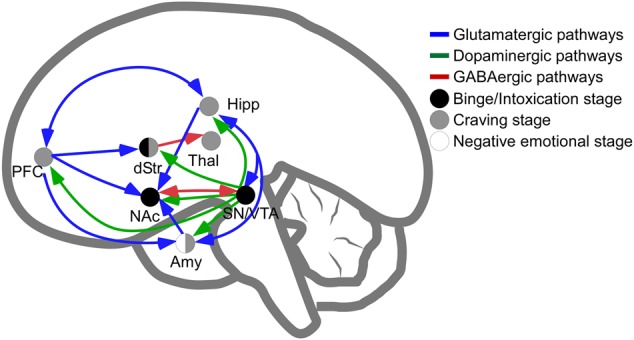
Schematic representation of pathways involved in intoxication, withdrawal and craving stages of addiction. Release of dopamine in the nucleus accumbens is a common feature of psychostimulants reinforcement at initial stages of psychostimulants intake (dots in black are regions directly involved in binge and intoxication). The negative emotional stage of withdrawal appears to be related to the activation of amygdala (dots in white are regions directly involved in negative emotional stage) and, finally, the latter stage of psychostimulant addiction, craving, depends on prefrontal cortex, amygdala and hippocampal activities (dots in gray). In blue, glutamatergic pathways; in green, dopaminergic pathways; in red, GABAergic connections. NAc, Nucleus Accumbens; Amy, Amygdala; dStr, dorsal Striatum; Hipp, Hippocampus; PFC, Prefrontal Cortex; SN/VTA, Substantia Nigra and Ventral Tegmental Area; Thal, Thalamus.

**FIGURE 2:**
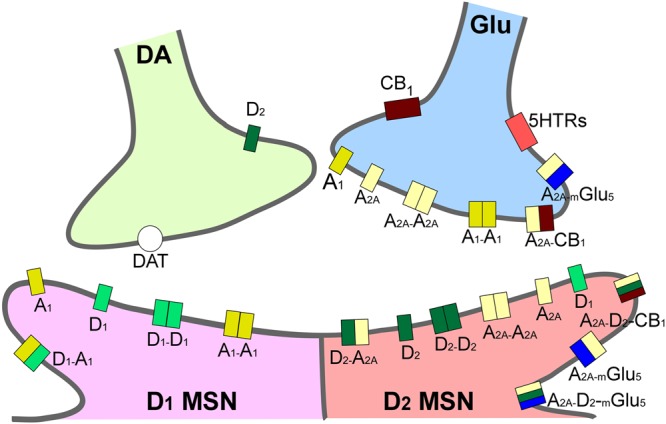
Integrative scheme of reward circuit in striatum with focus on adenosine and dopamine receptors and their interactions. A_1_ and A_2A_ receptors are located pre- and post-synaptically forming homo-, heterodimers and oligomers in the dendritic spines of medium spiny neurons in striatum (MSN). Glutamatergic input from cortex and dopaminergic input from ventral tegmental area project to both MSNs expressing D_1_-like and MSNs expressing D_2_-like dopamine receptors. A_2A_, adenosine 2A receptors; CB_1_, endocannabinoid CB_1_ receptors; DAT, dopamine active transporters; D_1_, dopamine D_1_ receptors; D_2_, dopamine D_2_ receptors; mGlu_5_, metabotropic glutamate subtype 5 receptors; 5HTRs, serotonin receptors.

The main brain circuitry associated with addiction is distributed across multiple areas. This circuitry is associated with the three stages of the addiction cycle (Koob and Volkow, 2010). The reinforcing effects in an initial binge/intoxication stage are mediated by DA and opioid neurotransmission, and depend on modifications in the VTA and striatum (particularly NAc). The negative emotional stage of withdrawal may be due to activation of the amygdala with norepinephrine, dynorphin and corticotropin-releasing factor. The third stage, craving, depends on the prefrontal cortex, amygdala and hippocampus, and glutamate is the major neurotransmitter involved (reviewed in Kelley and Berridge, 2002; Koob and Volkow, 2010; Kim et al., 2017; **Figure 1**). The NAc acts as a hub of convergence from the different regions, and it has been considered a key element in the neuronal circuitry of drug addiction. This nucleus is composed of two distinct populations of medium spiny neurons (MSN) with different levels of dopamine D_1_ and D_2_ receptors and projections (direct and indirect basal ganglia pathways), which are positively and negatively coupled to cyclic adenosine monophosphate (cAMP)/protein kinase A (PKA) signaling, respectively. Striatonigral MSN (direct pathway) send substantia P and dynorphin projections to substantia nigra/VTA and globus pallidus interna, and are enriched with dopamine D_1_ receptors. Striatopallidal MSN (indirect pathway) send enkephalin projections to globus pallidus externa and are enriched with dopamine D_2_ receptors (Lobo, 2009). Taking together several studies using different approaches such as analysis of psychiatric disorders, transgenic mice, neuropharmacology and optogenetic techniques, it has been possible to postulate an integrative representation of the synaptic connections in NAc and the neurotransmitter systems (Silberberg and Bolam, 2015; **Figure 2**).

Adenosine acts in the central nervous system (CNS) as a neuromodulator, with DA neurotransmission being one of its targets. The modulation of dopaminergic activity is mediated by two main subtypes of AR, being the antagonist of DA receptors. Specifically, A_1_ receptors colocalize with D_1_ receptors, and A_2A_ receptors with D_2_ receptors in heteromeric complexes (Fuxe et al., 2010). Building on this rationale, the case of A_2A_ receptors is particularly important when we are studying the effects of drugs of abuse, for reasons that have been extensively reviewed previously (Hack and Christie, 2003; Brown and Short, 2008; Filip et al., 2012), and that we only briefly enumerate here. First, A_2A_ receptors are highly expressed in the striatum, a key brain nucleus for the reward circuitry, and A_2A_ are crucial receptors, modulating behavioral responses induced by drugs of abuse (Ferré et al., 2007); indeed, their genetic deletion in mice results in a selective decrease of locomotor responses to cocaine and amphetamine (Chen et al., 2000). Furthermore, A_2A_ receptors are able to form heteromers with other adenosine subtypes, resulting in, e.g., the A_1_/A_2A_ heteromer (Ferré et al., 2008a), and also with families of receptors relevant for the treatment of some neuropsychiatric disorders and drug addiction, producing, e.g., A_2A_/mGlu_5_ (Ferré et al., 2002), A_2A_/D_2_ (Ferré et al., 2008b; Bonaventura et al., 2015), and A_2A_/CB_1_ receptor heteromers (Tebano et al., 2012). This spectrum of interaction opens a wide range of possibilities in the field of AR pharmacology (Casadó et al., 2009). Interestingly, the oligomer formed by A_2A_/mGlu_5_/D_2_ receptors confers to the A_2A_ subtype the ability to modulate the effects of psychostimulants in striatal neurons, through the balance of GABAergic, dopaminergic and glutamatergic signaling (Cabello et al., 2009; Kniazeff et al., 2011). The modulation of dopaminergic and glutamatergic signaling by A_2A_ receptors has been particularly important in the field of psychiatric disorders, as DA and glutamate are two key players in the processing of moods, which could also be very relevant in drug addiction-related disorders (Cunha et al., 2008).

## Adenosine Receptors Overview

### Adenosine Metabolism and Adenosine Receptors Structure

In the CNS, extracellular adenosine exists in basal conditions, and its concentration may increase under pathological situations, including hypoxia, ischemia or cell injury. Adenosine is produced by different mechanisms, including metabolism of ATP released from neurons or glial cells. Specifically, ATP undergoes dephosphorylation to ADP and AMP by the activity of particular enzymes named ectonucleoside triphosphate diphosphohydrolase (CD39), and to adenosine through a specific ecto-5′-nucleotidase (CD73) enzyme (Drury and Szent-Györgyi, 1929). Alternatively, adenosine may derive from hydrolysis of intracellular AMP through a cytoplasmic 5′-nucleotidase, or S-adenosyl-homocysteine (SAH) by SAH hydrolase, and may be released through facilitated diffusion, using bi-directional equilibrative nucleoside transporters (ENT) (Dunwiddie, 1985; Ledent et al., 1997; Ribeiro, 1999; Hack and Christie, 2003). Under resting conditions, extra- and intra-cellular levels of adenosine are very similar, but in pathophysiological states (inflammation, ischemia, and hypoxia), characterized by high levels of this nucleoside, transport through ENTs is the main mechanism responsible for extracellular adenosine removal. Inside the cell, adenosine is deaminated to inosine through adenosine deaminase (ADA), or phosphorylated to AMP by adenosine kinase (AK). These enzymes display different affinities for adenosine, with AK more affine than ADA; thus in physiologic conditions adenosine is preferentially transformed to AMP, whilst in pathological states predominantly to inosine, a process which occurs also in the extracellular milieu (Godinho et al., 2015; Borea et al., 2016).

Adenosine affects several functions in the body, exerting its physiological effects through regulation of four G-protein coupled receptors (GPCRs) named A_1_, A_2A_, A_2B_, and A_3_, characterized by different affinities for adenosine, tissue distribution, and coupling with effector systems. They have been cloned and pharmacologically characterized in different species, presenting a sequence homology of around 80–95%, except for A_3_ receptors which vary depending on species and show a variance of 30% in amino-acid composition between human and rat. The A_3_ receptor, in contrast to other AR, was the first to be isolated and then pharmacologically characterized (Meyerhof et al., 1991). All AR show a common structure, characterized by seven transmembrane domains connected by three intracellular and extracellular domains (Fredholm et al., 2000). At the extracellular level, the N-terminus presents specific glycosylation sites, while at the intracellular side, the C-terminus contains phosphorylation and palmitoylation sites, important for receptor desensitization. The A_2A_ receptor has a longer C-terminus tail constituted by 122 amino acids, whereas A_1_, A_2B_, and A_3_ receptors’ C-terminal domains comprise 30–40 amino acids (Fredholm et al., 2001). Recently, important crystallization results have determined the structures of human A_1_ and A_2A_ receptors, thus allowing better drug design for A_1_ and A_2A_ receptor-selective ligands (Jaakola et al., 2008; Lebon et al., 2011; Xu et al., 2011; Carpenter et al., 2016; Glukhova et al., 2017; Sun et al., 2017). In the case of A_3_ receptors, structure-based molecular modeling techniques have led to the rational design of potent A_3_ receptor-selective ligands (Ciancetta and Jacobson, 2017). Furthermore, AR may be present in the cell membrane in homomer isolated forms or in heteromers and oligomers, providing another possibility of intervention for drug development (Cabello et al., 2009). Specifically, the ability of AR to interact with many other receptors, such as A_2A_/D_2_ receptor heterodimers located in the striatum (represented in **Figure 2**), means they play a pivotal role in the modulation and integration of neurotransmission, and may be targeted by drugs for the treatment of neurological diseases, including drug addiction (Chen et al., 2013).

### Adenosine Receptors: Distribution, Signal Transduction, and Function

AR are widely distributed in almost all organ and tissues, spanning brain, heart, lung, liver, kidney, bone, eye, skin, joints, and blood cells, suggesting that these proteins are potentially able to affect almost every physiological function (Peleli et al., 2017).

Specifically, as for the presence of each AR subtype in CNS, the A_1_ receptor is mainly present in the cortex, hippocampus, cerebellum, nerve terminals, spinal cord, and glia (Chen et al., 2013). This wide range of locations reflects the multitude of physiological effects orchestrated by it, including inhibition of neurotransmitter release, reduction of neuronal excitability, sedation, anticonvulsant and anxiolytic effects, analgesia and regulation of sleep (Stenberg et al., 2003; Gessi et al., 2011; Sawynok, 2016; Vincenzi et al., 2016a,b; Varani et al., 2017). These effects are mediated through A_1_ receptors coupling to G_i_/G_o_ proteins, inhibition of AC, activation of phospholipase-C (PLC)β and, particularly in neurons, activation of potassium channels and deactivation of Q-, P-, and N-type Ca^2+^ channels. They also modulate mitogen-activated protein kinases (MAPK) with important functional effects (Schulte and Fredholm, 2003).

The A_2A_ receptor is highly expressed in the striatum, mainly present in GABAergic striatopallidal neurons, corticostriatal glutamatergic terminals, and cholinergic interneurons, but it is also detectable in the olfactory tubercle, cerebral cortex, hippocampus, neurons, and glial cells, where it induces excitotoxicity by affecting release of glutamate, activation of glia and infiltration of immune cells from the periphery, through blood brain barrier passage (Fuxe et al., 2003, 2007b; de Lera Ruiz et al., 2014). The A_2A_ receptor generally couples to G_s_ proteins to increase cAMP levels, but in the brain it stimulates G_olf_, a specific G_s_ protein in neurons also associated with AC (Kull et al., 2000), which is supposed to have a prominent role as a mediator of the locomotor effects of some psychostimulant drugs (Hervé et al., 2001). The signaling cascade starting from cAMP and PKA regulates different proteins such as cAMP responsive element binding protein (CREB) and DA- and cAMP-regulated phosphoprotein (DARPP-32) (Preti et al., 2015), which is also involved in the responses to psychostimulant drugs (Engmann et al., 2015). CREB phosphorylation then increases transcription of immediate early genes such as c-fos and other genes like preproenkephalin (Ferré, 2008). In addition, the A_2A_ receptor, with its long C-terminus, could also bind to different accessory proteins like D_2_ receptors, ADP-ribosylation factor nucleotide site opener (ARNO), α-actinin, translin-associated protein X (TRAX) and ubiquitin-specific protease (USP4). A_2A_ receptor activation may also trigger the Ras/Raf-1/MEK/ERK pathway through PKA-dependent or independent mechanisms (Schulte and Fredholm, 2003).

A_2B_ receptors are present in astrocytes, neurons, and microglia, but their role in the CNS is less well characterized in comparison to the other AR subtypes. As for the effector systems, it activates G_s_ proteins/cAMP/PKA phosphorylation. Furthermore, the A_2B_ receptor stimulates an increase in G_q_ protein/PLC/Ca^2+^, while modulating ion channels through βγ subunits. Coupling to MAPK has been also reported (Merighi et al., 2017).

Finally, for A_3_ receptors, a low level of expression has been detected in the brain, in which it was detected in the cortex, thalamus and hypothalamus, hippocampus, motor nerve terminals, retinal ganglion cells, pial and intracerebral arteries and glia (Borea et al., 2015; Jacobson et al., 2017). The A_3_ receptor couples to G_i_ proteins and decreases cAMP levels, whilst through G_q_ proteins or G_βγ_ subunits, it stimulates PLC and increase Ca^2+^ concentration. In addition, a pathway presenting RhoA, a monomeric G-protein and PLD, is relevant for the neuroprotection effects of A_3_ receptors. Inhibition of the transcription hypoxia-inducible factor (HIF-1) has been reported in astrocytes with neuromodulatory effects through MAPK and Akt modulation (Gessi et al., 2013). Interestingly, the reduction of neuroinflammation has been related to analgesia (Janes et al., 2014).

## Psychostimulants and Adenosine Receptors

In this section, we will describe the current state of knowledge about how adenosine signaling can interfere with common addictive psychostimulant consumption, focusing on data obtained from animal, particularly murine, models and from human studies. Animal models not only give us useful information on the pathophysiological mechanisms of psychostimulant drugs intake that are not accessible to study in human subjects but also provide an useful tool to assay pharmacological approaches to explore potential treatments before develop clinical trials. Certainly, although animal models may produce similar responses to those observed in humans, all the paradigms used in them for drug addiction research imply a lower degree of complexity than that observed in human drug addiction. Nowadays, more complex animal models have been developed to include some behavioral responses observed in human addictions, such as social peer influences in drug intake (Moser et al., 2011; Ross et al., 2015; Strickland and Smith, 2015). Nevertheless, in the last part of this section, information obtained from human studies is also provided and the actual information regarding this issue is discussed.

Psychostimulants can be classified into two broad categories depending on the mechanism by which DA levels are increased; namely, amphetamines (AMPH) behave as DA releasers, while cocaine acts to inhibit DA reuptake trough the inhibition of the DA active transporter (DAT) (Siciliano et al., 2015). In addition to the increase in levels of DA in the striatum, AMPH and cocaine are able to induce an increase in norepinephrine (NE), by blocking NE transporter (NET), while only cocaine also increases serotonin, by inhibiting serotonin transporter (SERT) (Phillips et al., 2014; Zwartsen et al., 2017).

Cocaine is extracted from coca leaves, mainly in South America where the coca plant is commonly grown. Despite being the most widely used illicit narcotic drug, cocaine has been used for centuries (if not millennia) for medical and cultural purposes (Johanson and Fischman, 1989). Although cocaine may be illegally distributed in several forms, mainly cocaine hydrochloride but also cocaine sulfate or crystalized as “crack,” the physiological and psychoactive effects of cocaine in different forms are similar (Hatsukami and Fischman, 1996).

The family of “amphetamines” or amphetamine-like psychostimulants includes a wide range of compounds which can be synthetized based on chemical substitutions of the original structure of alpha-methylphenethylamine. AMPH and methamphetamine (S(+)-methylamphetamine, METH) are the most studied compounds of the family, but other well-known psychostimulants in this family include methylphenidate, MDMA (3,4-methylenedioxymethamphetamine, “ecstasy”), ephedrine and cathinone (synthetic derivatives of which are known as “bath salts”) (Sulzer et al., 2005). Specifically, although cathinones have a synthetic profile similar to AMPH, their mechanism of action is rather similar to that of cocaine, being potent DAT inhibitors (López-Arnau et al., 2017). On the other hand, MDMA, in addition to inhibiting NET and DAT, is both a substrate for SERTs and an inhibitor of them, with an IC_50_ in the micromolar range, thus differentiating its behavior from that of AMPH, which is unable to affect SERTs (Rudnick and Wall, 1992; Baumann et al., 2005; Zwartsen et al., 2017). Although AMPH and METH have similar pharmacokinetics, they differ in their pharmacodynamic properties, with METH inducing DA release in the NAc more efficiently than AMPH (Goodwin et al., 2009). AMPH and METH have been studied for a long time, but their neurobiology remains largely unknown with discrepancies in the literature between pharmacological and genetic-based experiments.

As for psychostimulants-induced changes in AR expression in brain areas only few scientific reports have been reported. Specifically, during a study of cocaine withdrawal and its relationship with sleep architecture, Yang et al. (2011) reported that A_1_ receptor expression in the hippocampus of rats was reduced after 14 days of withdrawal, A_2A_ receptor density was increased on withdrawal-day 8 and 14, while the A_2B_ receptors remained unchanged. Other findings provide neurochemical evidence that after 10 days of cocaine self-administration, an up-regulation of functional A_2A_ receptors in the NAc of rats was induced that returned to baseline expression levels after 7 days of drug withdrawal (Marcellino et al., 2007). In a study of the motivational mechanisms after cocaine self-administration and extinction, it was reported that, in the dorsal striatum of Wistar rats, there was an increase in the affinity of A_2A_ receptors during maintenance and an increase in A_2A_ receptor density after extinction from cocaine self-administration (Frankowska et al., 2013). Nevertheless, using a paradigm of escalating administration of cocaine dose (“binge”) and subsequent withdrawal, the density of adenosine A_1_ and A_2A_ receptors in various brain nuclei of Fischer rats was not different nor in chronic cocaine-treated rats or in the long-term withdrawn rats group (Bailey et al., 2005). Finally, rats trained to self-administer METH for 14 days showed selective altered expression of AR, with A_1_ receptor levels increased in the NAc shell, caudate-putamen and prefrontal cortex, and A_2A_ receptors decreased in the NAc shell and raised in the amygdala (Kavanagh et al., 2015). Interestingly, it is well known that GPCR act as an oligomer, and indeed homodimers of A_2A_ and 5-HT1A receptors occur constitutively, and are further increased by agonists such as CGS 21680 and 8-OH-DPAT, or reduced by antagonists including SCH 58216 and methysergide, which could also contribute to psychostimulant addiction (Łukasiewicz, 2007).

Even though there are no consistent and complete studies about amphetamines- and cocaine-induced changes in AR expression in brain areas, a prominent role in the modulation of psychostimulant addiction attributed to adenosine is mediated through the activation of AR by complex mechanisms, affecting various aspects of this phenomenon including locomotor activity, discrimination, seeking behavior and reward.

### Studies in Animal Models

Although the interactions between adenosine and DA in the striatum were previously known, the role of AR in AMPH-induced locomotor responses was first characterized only at the end of the last century. Turgeon et al. (1996) demonstrated that, in Sprague-Dawley rats, AMPH-induced behavior could be pharmacologically modulated by pretreatment with CHA, an A_1_ receptor agonist, or APEC, an A_2A_ receptor agonist, which reduced locomotor responses induced by acute AMPH exposure. However, only the A_2A_ receptor agonist inhibited c-Fos immunoreactivity, induced by AMPH, in striatum and NAc. In the case of METH, the experimental paradigm used to determine the role of AR in METH-mediated effects was METH-induced toxicity. In these studies, administration of the A_1_ agonist CPA attenuated the METH-provoked neurochemical tyrosine hydroxylase changes in Swiss-Webster mice (Delle Donne and Sonsalla, 1994) while, in other experimental models, represented by Wistar rats, both CPA and CGS 21680, an A_2A_ receptor agonist, were able to attenuate METH-mediated DA release in the striatum (Gołembiowska and Zylewska, 1998a). In terms of METH-induced locomotor responses, it was also reported that administration of CHA and CGS 21680 before acute METH exposure in Wistar rats was able to inhibit METH-induced hyperlocomotion (Shimazoe et al., 2000) but, interestingly, when those same agonists were tested to study METH-induced sensitization (which occurs after repeated intermittent drug administration), only CGS 21680 was able to inhibit METH-induced increase of locomotion while CHA had no effect (Shimazoe et al., 2000). In addition, the activation of A_2A_ receptors could also be the mechanism by which some herbal compounds, PAP9704 and ginsenoside herbal compounds, attenuate METH-induced hyperlocomotion and conditioned place preference in BALB/C AnNcrj mice as well as in C57BL/6 mice, respectively (Kwon et al., 2004; Shin et al., 2005). Furthermore, AMPH-induced stereotyped head movements in Wistar rats were attenuated in a dose-dependent manner with CGS 21680, poorly reduced when CPA was used and even potentiated when DMPX, an A_2_ receptor antagonist, was used (Poleszak and Malec, 2000). Finally, it seemed that the inhibition of AMPH-induced stereotyped head movements, through activation of A_1_ receptor, could depend on agonist properties, as Ribavirin, an A_1_ receptor agonist, reduced AMPH-induced total locomotor activity but had no effects on stereotypic activity in Wistar rats (Janać et al., 2005). Relevant literature concerning the functional effects of AR ligands in psychostimulant-induced phenomena, with a focus on rodent models, are presented in **Supplementary Table S1**.

A complete study of AR and their relationship with cocaine-induced locomotion was carried out by Poleszak and Malec (2002b) at the beginning of this century. They reported that CPA and CGS 21680, decreased both cocaine- and AMPH-induced locomotor activity. The agonist doses required to inhibit the effect of AMPH were higher than those which were active in cocaine-induced hyperactivity, while the A_2_ antagonist DMPX enhanced the effects of AMPH in Swiss mice (Poleszak and Malec, 2002b). Accordingly, the selective stimulation of A_2A_ receptors in Wistar rats using CGS 2160 reduced the cocaine-induced locomotor response, the locomotor response during the development of sensitization, and the expression of sensitization in a cocaine challenge dose, while blocking A_2A_ receptors with the antagonist MSX-3 induced the opposite effects in the three studied paradigms (Filip et al., 2006).

Genetic deletion of A_2A_ receptors (A_2A_ KO) in animal models of drug addiction provides a tool to understand the role of these receptors under certain circumstances by comparison to wild-type animals. Nevertheless, the results obtained using A_2A_ KO animals on cocaine-, AMPH-, and METH-induced behavioral responses seem contradictory. In this sense, it was reported that in 129-Steel and hybrid C57BL/6 × 129-Steel mice, A_2A_ KO attenuated cocaine-induced locomotor stimulation (Chen et al., 2000). Similar experiments, performed to demonstrate the effects of A_2A_ genetic deletion with independence of the genetic background, were reproduced later in pure 129-Steel mice resulting in missed AMPH-induced locomotor sensitization (Chen et al., 2003). Accordingly, similar results were obtained when hybrid C57BL/6 × 129-Steel animals were used to generate tissue-specific A_2A_ KO animals, where deletion of forebrain A_2A_ receptors was carried out, which showed a loss of AMPH-mediated locomotor response (Bastia et al., 2005). In contrast, in CD1 background mice, it was reported that there were no differences in the cocaine-induced locomotor activity, sensitization and conditioned place preference between A_2A_ KO animals and their littermates (Soria et al., 2006). Authors only found a lower rate of cocaine self-administration and motivation as well as lower efficacy of cocaine reinforcing effects in A_2A_ KO mice (Soria et al., 2006). Interestingly, Soria et al. (2006) concluded with the hypothesis that separate neuronal substrates could mediate cocaine-induced locomotor effects and self-administration in an operant behavior paradigm. In order to corroborate this hypothesis, Shen et al. (2008) designed two different KO animals to distinguish between striatal versus non-striatal cocaine-mediated effects. In these experiments, cocaine-induced locomotor activity was enhanced in striatum-specific A_2A_ KO mice (A_2A_ receptors were deleted in striatal neurons) but attenuated in forebrain-specific A_2A_ KO mice (A_2A_ receptors were deleted in the neurons of striatum, cerebral cortex, and hippocampus). In addition, pharmacological inactivation (using KW6002, an A_2A_ receptor antagonist with preferential affinity for post-synaptic A_2A_ binding sites) of extra-striatal A_2A_ receptors in striatum-specific A_2A_ KO mice attenuated cocaine-induced hyperlocomotion, while the same antagonist enhanced cocaine-induced hyperlocomotion in the wild-type mice, reflecting the antagonism between striatal A_2A_ receptors and extra-striatal A_2A_ receptors (Shen et al., 2008). Finally, in CD1 A_2A_ KO mice, a lesser increase in DA levels after acute cocaine exposure was reported while locomotor activity was further increased in A_2A_ KO mice in comparison to wild-type littermates (Wells et al., 2012). The genetic models derived from the manipulation of AR, and their effect on the interaction of AR with psychostimulants, are presented in **Supplementary Table S2**.

Interestingly, there is some research available about the role of A_3_ receptors in modulation of AMPH- and METH-mediated actions. METH-induced DA release was measured in the rat striatum using APNEA, a putative A_3_ receptor agonist, which has a biphasic effect when perfused locally to the striatum via microdialysis. At the lower concentration studied, APNEA induced a decrease in DA outflow, but at the higher concentration studied, a clear increase in DA outflow was reported, which led researchers to conclude that the activation of A_3_ receptors exerts a rather toxic effect on DA neurons (Gołembiowska and Zylewska, 1998b). Nevertheless, when the A_3_ receptor was genetically deleted, it was reported that the resultant mice were much more sensitive to the toxic actions of METH, including Iba-1, caspase 3, TNF-α, and vesicular monoamine transport 2 (VMAT) increased expression (Shen et al., 2011), and also presented reduced AMPH-induced locomotor response (Björklund et al., 2008).

Adenosine receptors modulate psychostimulant-induced discriminative-stimulus effects, as A_1_ and A_2A_ receptors antagonists (CPT and MSX-3 or DMPX, respectively) partially mimicked the discriminative-stimulus effects of METH, by increasing the levels of drug-lever selection, and potentiating the discriminative-stimulus actions of METH, as shown by significant leftward shifts of the METH dose-response curve, behaving like psychostimulant drugs (Munzar et al., 2002; Justinova et al., 2003). Surprisingly, CPA and CGS 21680 also shifted the dose-response curve to the left for cocaine, but not for METH, suggesting that A_1_ and A_2A_ receptors have different influences on the discriminative-stimulus effects of METH and cocaine in Sprague-Dawley rats (Justinova et al., 2003).

Another relevant aspect in drug addiction which is strongly influenced by AR is the seeking behavior. In terms of the effect of A_1_ agonists, CPA microinfusions in the NAc of Sprague-Dawley rats inhibited cocaine seeking behavior (Hobson et al., 2013). On the other hand, treatment with A_2A_ agonists such as NECA or CGS 21680 reduced the number of cocaine infusions self-administrated by rats, mainly due to an increase in the latency for the first cocaine infusion (Knapp et al., 2001). Accordingly, the activation of A_2A_ receptors, using CGS 21680, antagonized the reinstatement of cocaine seeking in Sprague-Dawley rats (Bachtell and Self, 2009). In addition, it was reported that A_2A_ receptor blockade, using CGS15943, increased cocaine-seeking in a dose-dependent manner and also reinstated cocaine-seeking, functioning as an intravenous reinforcer, in baboons (Weerts and Griffiths, 2003). The effects of activation and blockade of A_2A_ receptors, using CGS 21680 and MSX-3, respectively, were also tested in Sprague-Dawley rats trained to press a lever for cocaine. Pretreatment with intra-NAc core microinjections of CGS 21680 reduced cocaine-induced reinstatement, while MSX-3 exacerbated it (O’Neill et al., 2012). Similar results were obtained in Sprague-Dawley rats, where intra-NAc microinjections of CPA and CGS 21680 inhibited the expression of cocaine sensitization, and microinjections of ABT-702 and DCF (AK and ADA inhibitors, respectively) blocked cocaine sensitization (Hobson et al., 2012). Interestingly, A_2A_ receptor activation (with CGS 21680) in Wistar rats was able to affect food seeking with a similar potency to that observed for cocaine seeking, whilst A_2A_ receptor antagonists increased cocaine-, but not food-, seeking behavior, suggesting that possibly a differential expression of A_2A_ receptors occurs in striatopallidal GABAergic neurons involved in cocaine and food seeking (Wydra et al., 2015). In contrast, it was reported that although the activation of A_1_ (using CPA) and A_2A_ (using CGS 21680) receptors impaired initial extinction responding, the blockade of presynaptic A_2A_ receptors using SCH 442416 produced persistent impairment of cocaine-induced seeking in Sprague-Dawley rats (O’Neill et al., 2014).

As reported in **Supplementary Table S1**, the vast majority of studies have focused exclusively on males. Nevertheless, there is a large amount of literature concerning the differences in drug-mediated effects between males and females (for a recent review see Lynch, 2017). Although the focus of the present review is on the relationship between AR and psychostimulants, sex differences will be briefly commented upon to provide a fuller picture of the problem of addiction. In this regard, it has been reported that female rats self-administer higher levels of cocaine or METH, and escalate intake faster than males during extended daily psychostimulant access (Roth and Carroll, 2004; Reichel et al., 2012). Females also require shorter periods of time to show increased motivation to obtain cocaine (Lynch and Taylor, 2004). In addition, following short access self-administration, females show markedly higher levels of METH seeking (Ruda-Kucerova et al., 2015), and females present increased cocaine-seeking behavior during cocaine withdrawal (up to 6 months) compared to males (Kerstetter et al., 2008). Sex-dependent responses to AR’s ligands have also been reported in Sprague-Dawley rats using ATL444, a novel A_2A_/A_1_ receptor antagonist, in studies of motivation for cocaine. In these experiments, it was reported that ATL444 treatment acutely increased motivation for cocaine in females but, in males, induced a long-term decrease in motivation for cocaine (Doyle et al., 2012). Finally, one interesting more recent study, evaluating the effects of A_2A_ receptor deletion on schizophrenia, found that AMPH induced a lower hyperlocomotion response in male CD1 A_2A_ KO mice at 120–170 min, in comparison to wild type AMPH-treated mice; this effect was observed to a major extent in female CD1 A_2A_ KO mice at 70–180 min, in comparison to wild type AMPH-treated mice (Moscoso-Castro et al., 2016).

Rewarding effects induced by AMPH and METH have mainly been evaluated using conditioned place preference procedures. In this way, Poleszak and Malec (2003) proved that CPA and, under certain conditions, CGS 21680, reduced the development of AMPH-induced conditioned place preference in Wistar rats; however, only CGS 21680 was able to decrease the expression of METH-induced conditioned place preference. In addition, ginseng saponins reduced the METH-induced circling behavior and conditioned place preference in C57BL/6 mice, via activation of the A_2A_ receptor, as this reduction was reversed in a dose-dependent manner using the A_2A_ receptor antagonist CSC. Interestingly, reduction of AP-1 DNA binding activity and proenkephalin gene expression induced by METH exposure were reduced by CSC (Shin et al., 2005). Furthermore, C57BL/6J mice with D_2_ receptors knocked down in the NAc core have been reported to exhibit a reduction in METH-induced locomotion, as in other paradigms (locomotor sensitization and conditioned place preference) after repeated METH-treatment, suggesting that D_2_ receptors are necessary mediators for the development of METH-induced rewarding effects (Miyamoto et al., 2014). Thus, the antagonism between A_2A_ and D_2_ receptors further supports the conclusion that the activation of A_2A_ receptors could be a promising way to counteract AMPH- and METH-induced rewarding effects.

A series of experiments to increase our understanding of the role of A_1_ and A_2A_ receptors in METH-induced behavior were designed by Kavanagh et al. (2015), reporting that the initial METH-mediated rewarding effects may be tempered by A_1_ or A_2A_ receptor activation in a model of rat self-administration. Therefore, they found that in Sprague-Dawley rats, the stimulation of A_1_ receptors using CPA reduced METH self-administration, and that the stimulation of both A_1_ and A_2A_ receptors (using CPA and CGS 21680, respectively) reduced METH-induced place preference (Kavanagh et al., 2015). These results suggest that, taking into account the antagonism of A_1_/D_1_ and A_2A_/D_2_ heteromers, both A_1_ and A_2A_ agonists will be useful to reduce METH-induced behaviors during the initial exposures to METH but, when METH exposures are more prolonged, the modulation of AR renders only the A_1_ agonist powerful enough to counteract the rewarding properties of METH. Accordingly, A_2A_ KO animals (CD1 background) were less sensitive to METH rewarding properties, as METH exposure did not induce conditioned place preference in those animals and, although METH-self administration was not altered, the motivation to self-administer METH was reduced when compared with wild-type (Chesworth et al., 2016).

Finally, the important role of AR as possible pharmacological tools to treat psychostimulant addiction has also been tested in animal models using other members of the amphetamine family, albeit to a lesser extent. Specifically, it was demonstrated that SCH 58261, but not DPCPX, increased MDMA-induced hyperthermia (Vanattou-Saïfoudine et al., 2010) but, conversely, DPCPX, but not SCH 58261, enhanced MDMA-induced DA release from striatal slices (Vanattou-Saïfoudine et al., 2011). Accordingly, the blockade of A_1_ or A_2A_ receptors using DPCPX or KW 6002, respectively, in mouse striatum increased the MDMA-mediated release of DA and 5-HT (Górska and Gołembiowska, 2015). In contrast to these pharmacological experiments, when A_2A_ receptors were knocked down in a CD1 background model, MDMA-mediated reinforcement was dramatically decreased (although locomotor response was not altered) compared to wild-type littermates (Ruiz-Medina et al., 2011), suggesting that the lack of A_2A_ receptors will increase resistance to psychostimulant rewarding properties.

### Human Studies

The genomic era has provided the opportunity to study human polymorphisms and so to provide a tool to design personalized treatments according to observed mutations. Recent meta-analysis of case-control studies of psychostimulant users (cocaine, AMPH, and METH) have revealed that there is a general down-regulation of the dopaminergic system, as in psychostimulant users there is a decrease in DA release, in DA transporter availability, and also in the levels of D_2_ and D_3_ receptors, concluding that DA function is down-regulated both pre- and post-synaptically. This suggests that restoring DA function must be an important goal in the treatment of psychostimulant abusers (Ashok et al., 2017). Due to the antagonistic relationship between dopaminergic function and AR, some authors have studied the relationship between A_1_ and A_2A_ gene polymorphisms and susceptibility to psychostimulant consumption/addiction. Most relevant human studies are presented in **Supplementary Table S3**.

The first study designed to discern the influence of A_1_ and A_2A_ gene (ADORA1 and ADORA2A, respectively) polymorphisms on inter-individual variability in AMPH response was carried out by Hohoff et al. (2005). Using a sample of 99 healthy volunteers (50 men and 49 women), who received AMPH or a placebo, the authors reported that two ADORA2A polymorphisms (1976C/T and 2592C/T_ins_) were associated with increases in reported anxiety by participants after AMPH consumption (Hohoff et al., 2005). Nevertheless, these results should be regarded with caution, as the same research group could not reproduce them using the same methodology for a larger sample of individuals (Hart et al., 2013). In addition, a study by Kobayashi et al. (2010) searching for the relationship between ADORA2A variations and susceptibility to METH dependence/psychosis reported that, in a population of 171 Japanese METH dependent/psychotic patients (compared to 229 control subjects), six ADORA2A polymorphisms were found. The authors reported that only one single nucleotide polymorphism (SNP) of the A_2A_ receptor gene was significantly associated with a subgroup of female patients (METH dependent/psychotic) that consumed only METH and no other psychostimulants or drugs (Kobayashi et al., 2010). Interestingly, that SNP was 1976C/T (rs5751876), the same that Hohoff et al. (2005) associated with anxiety after AMPH consumption, and this SNP is a synonymous variant, meaning that it cannot include amino acid substitutions. Finally, in the same Japanese population as the previous study, seven ADORA1 SNPs were identified but none was specific to any subgroup of METH dependent/psychotic patients, which would suggest that ADORA1 polymorphisms would make little or no contribution to METH vulnerability (Kobayashi et al., 2011); however, further research is needed to confirm this supposition. In addition, caffeine-induced anxiety has also been associated with ADORA2A 1976C/T polymorphism in a sample of 102 individuals (Childs et al., 2008), although other ADORA2A polymorphisms (such as 1976TT) also seem to be related to caffeine-induced anxiety, and could also influence predominantly women vulnerable to anxiety (Domschke et al., 2012; Gajewska et al., 2013).

Despite the huge amount of evidence that connects AR with psychostimulant-mediated actions, the translation of this knowledge to the clinic has been quite slow in comparison with other areas. A few reasons related to particular characteristics of AR could be that AR receptors are widely distributed, not only in the CNS but throughout the human body, with adenosine signaling responsible for the regulation of a broad spectrum of physiologic and pathologic actions (for a more detailed discussion see Müller and Jacobson, 2011; Chen et al., 2013). For this reason, it is experimentally difficult to demonstrate the clinical effectiveness and safety of an AR ligand. Therefore, only two clinical studies (registered in website^1^) have studied psychostimulant dependence and its link with AR (**Supplementary Table S3**). One of these compared the responses of volunteers to acute caffeine (150 and 300 mg), AMPH (20 mg) and placebo between 13 cocaine users and 10 healthy control subjects (NCT00733993). The main target of the trial was to study caffeine-mediated effects in cocaine users. Nevertheless, although caffeine and AMPH produced a series of differential results across the cocaine and control groups, these outcomes were not systematic, perhaps due to limitations of the study itself (Lane et al., 2014). On the other hand, the effect of an acute dose (100 mg) of the A_2A_ antagonist SYN115 was studied to elucidate the effects of this antagonist on brain function and behavior in a group of cocaine-dependent volunteers (NCT00783276). Some subjective effects (consistent with stimulation) were induced by SYN115 administration in cocaine users (Lane et al., 2012). Furthermore, the administration of SYN115 to cocaine-dependent volunteers increased brain activation in the orbitofrontal cortex, insula, and superior and middle temporal pole, as measured by fMRI while the participants were performing working memory tasks; this suggests that the blockade of A_2A_ receptors could mitigate cocaine-associated neurobehavioral deficits (Moeller et al., 2012). In addition, no clinically significant adverse cardiovascular events were reported by the volunteers in either study (Lane et al., 2012; Moeller et al., 2012).

Finally, epidemiological and preclinical data demonstrate that gender differences exist for the three phases of drug abuse (represented in **Figure 1**). The pattern of gender differences establishes that women have lower prevalence of drug use disorders involving both licit and illicit drugs (including alcohol, sedatives, cannabis, tranquilizers, opioids, hallucinogens, and cocaine use disorders). Nevertheless, women that begin to self-administer drugs, even at lower doses than men do, escalate faster to addiction and present higher rates of relapse compared to men. These gender differences can be interpreted in terms of sociocultural factors as well as biological/physiological factors (reviewed in Lynch, 2006; Lev-Ran et al., 2013; Bobzean et al., 2014; Becker and Koob, 2016). Men and women also differ markedly in terms of psychostimulant use/abuse. For example for METH consumption, women tend to begin METH use at earlier ages and seem more dependent on METH consumption than men, although women do suffer a decreased degree of toxicity and respond better to treatment (Dluzen and Liu, 2008). In addition, women present more severe problems related to cocaine intake, beginning to use cocaine at earlier ages, and some pharmacological treatments for drug addiction have poor outcomes among women compared to men (Kennedy et al., 2013; DeVito et al., 2014). Several pathophysiological studies have demonstrated that the reinforcing effect of cocaine is strongly influenced by the female hormonal cycle; in fact, some authors suggest that gender differences in addiction are due to differences in the reinforcement pathways of neural systems induced by ovarian hormones (Anker and Carroll, 2011; Bobzean et al., 2014). These findings highlight the importance of taking gender into account when analyzing psychostimulant use, and designing prevention programs and personalized treatment programs.

## Mechanisms of Adenosine Receptor-Mediated Pathways in Drug Addiction

As noted in previous sections, it has been reported that AR interact in an antagonistic way with DA receptors, A_1_ receptors being colocalized in heteromeric complexes with D_1_ receptors, and A_2A_ receptors with D_2_ receptors, counteracting DA-induced behavioral effects (Ferré et al., 1997; Ginés et al., 2000; Hillion et al., 2002; Fuxe et al., 2007a). Specifically, the stimulation of striatal D_2_ receptors is responsible for the locomotor, sensitizing and rewarding effects of drugs of abuse such as cocaine and amphetamines, and A_2A_ receptor stimulation counteracts them (Heffner et al., 1989; Popoli et al., 1994; Rimondini et al., 1997; Poleszak and Malec, 2000, 2002a; Shimazoe et al., 2000; Knapp et al., 2001; Bachtell and Self, 2009; Jastrzêbska et al., 2014; Johnson and Lovinger, 2016). In general, the process of addiction depends on an increase in DA neurotransmission in the striatum and an activation of its receptors. Specifically, cocaine induces its effects by indirectly increasing DA levels and directly activating D_2_ receptors (Fuxe et al., 2007a; Ferraro et al., 2012), thus enhancing dopaminergic signaling. The DA receptors most involved are of the D_2_ subtype, as demonstrated by their persistent striatal decrease following drug detoxification, and their induction of relapse as a consequence of chronic drug administration.

Interestingly, A_2A_ and D_2_ receptors are co-expressed in the striatum, forming heteroceptors, especially in the GABAergic striatopallidal neurons, where A_2A_ receptor activation increases GABA release and counteracts the effects induced by D_2_ receptors. These receptors may be linked to each other in two opposite ways. On the one hand, these receptor subtypes may form heteromers, causing antagonistic interactions between A_2A_ receptors and D_2_ receptors at the AC level, related to G_s/olf_ and G_i_ type V AC signaling. On the other hand, at the membrane level, A_2A_ receptor activation exerts a counterbalancing effect to D_2_ receptor stimulation, by reducing its affinity for DA and decreasing functional effects induced by D_2_ receptor stimulation (Ferré et al., 1991, 1994). In support of this relationship, transgenic animal models overexpressing A_2A_ receptors in the brain showed reduced D_2_ receptors in the striatum. Accordingly, A_2A_ receptor activation decreases behavioral responses to psychostimulants, indicating that the A_2A_ receptor may represent a novel drug target for the treatment of drug addiction. In particular, A_2A_ receptor stimulation decreases cocaine reward and seeking behavior, by reducing D_2_ agonist affinity (Pintsuk et al., 2016). In the context of the antagonistic interaction between A_2A_/D_2_ receptors, it has also been reported that the D_2_ receptor, through coupling to G_i_, inhibits A_2A_ receptor-mediated cAMP/PKA signaling, and thus CREB phosphorylation and c-fos expression (Pinna et al., 1997; Kull et al., 2000; Hillion et al., 2002). However, synergistic A_2A_ and D_2_ receptor interaction has been revealed, again at the AC level in the striatum, linked to the overexpression of activator of G protein (AGS3) and G_s/olf_ and G_i_ type II/IV AC pathway. This relationship becomes important when AGS3 is upregulated, such as during ethanol consumption, and withdrawal from cocaine, ethanol or morphine, because its activity stabilizes and inhibits the GDP-bound form of G_i_, at the same time increasing the βγ-dependent effect of G_s/olf_ protein, producing a strong increase in cAMP-PKA signaling. Even though in the striatum the first A_2A_/D_2_ antagonistic relationship is predominant, due to the higher distribution of AC V, when AGS3 is upregulated, such as during chronic exposure to addictive drugs, the synergistic interaction between A_2A_ and D_2_ receptors becomes relevant, suggesting that A_2A_ receptor antagonists may represent a class of drug to combat addiction and relapse (Ferré et al., 2008b). In addition, neuroprotection exerted by A_2A_ receptor agonists seemed to be mediated by an increase in nuclear factor-κB (Kermanian et al., 2012, 2013; Soleimani et al., 2012).

Furthermore, neuromodulation of neuronal networks by systemic A_2A_ receptor activation inhibits the reward and motivational properties of cocaine targeting A_2A_/D_2_ heteroreceptors in the striatopallidal GABA pathway. Microdialysis studies have related this effect to their increase and reduction of GABAergic and dopaminergic transmissions, respectively, in the NAc, as a consequence of an antagonistic A_2A_/D_2_ interaction, both at the membrane cell surface and at the intra-cellular level (Fuxe et al., 2007a; Trifilieff et al., 2011; Franco et al., 2013; Wydra et al., 2015; Borroto-Escuela et al., 2017). A_2A_/D_2_ heteromers involved in reward mechanisms reside in GABAergic neurons of the ventral striatopallidal area that are responsible for rewarding, motivational and seeking effects induced by cocaine, as well as by food (Wydra et al., 2013). However, both systemic treatment with an A_2A_ receptor antagonist and its direct injection into the NAc reduced relapse in heroin-addicted rats and prevented DA increases in the NAc shell induced by tetrahydrocannabinol (THC), but not those mediated by cocaine (Yao et al., 2006; Justinova et al., 2011). Furthermore, A_2A_ receptor antagonists alone may behave like psychostimulants by triggering cocaine-seeking behavior, thus decreasing their utility in the treatment of drug-dependence. Indeed, some findings on the addictive properties of A_2A_ receptor antagonists have reported that they substituted for cocaine in baboons (Weerts and Griffiths, 2003), also inducing conditioned place preference (Harper et al., 2006) and restored cocaine-seeking behaviors in rats (O’Neill et al., 2014). In addition, the blockade of A_2A_ receptors increased DA in the striatal network in cocaine-dependent subjects, which resulted in major prefrontal cortex stimulation (Moeller et al., 2012). However, in rats trained to self-administer heroin, the administration of A_2A_ antagonists eliminated reinstatement (Yao et al., 2006), opening the possibility of using A_2A_ antagonists as therapeutic ligands in the management of abstinence in the addiction of some drugs. The effect of cocaine exposure in fetal brains and the modulation of DA and adenosine effects have also been addressed; specifically, from E8 to E14 embryonic days, cocaine treatment induced changes in DA and adenosine signaling which increased basal cAMP levels in the striatum and cerebral cortex. This effect could be reverted by blocking A_2A_ receptors (using SCH58261), suggesting that A_2A_ receptors could be considered good candidates as targets to treat prenatal cocaine exposure-related syndromes. Indeed, D_2_ and A_2A_ receptors counterbalance each other’s effects in the embryonic brain in a similar manner to what happens in the mature brain (Kubrusly and Bhide, 2010).

A_2A_ receptors, independent of their interaction with D_2_ receptors in A_2A_/D_2_ heteromers, are also present in other complexes with mGlu_5_ and CB_1_ in striatal GABAergic neurons, as well as with A_1_, mGlu_5_, and CB_1_ in striatal glutamatergic terminals (**Figure 2**), that may be involved in the modulation of reward, exerting an important role in the regulation of dopaminergic and glutamatergic effects in addiction (Kalivas and Volkow, 2011; Cahill et al., 2014; Zhang et al., 2014; Johnson and Lovinger, 2016). In this sense, the blockade of A_2A_ receptors increased cocaine-mediated locomotor effects through the activation of CB_1_ receptors in rat striatum (Tozzi et al., 2012). It has also been demonstrated that an interaction between A_2A_ receptors and metabotropic glutamate 5 receptors (mGlu_5_) in the striatum avoided METH-, but not cocaine-, induced hyperactivity and rewarding behavior, making a combined antagonism of A_2A_ and mGlu_5_ receptors in the therapy of METH addiction possible (Wright et al., 2016). Accordingly, the influence of adenosine on glutamatergic transmission in the striatal region has been reported (Fuxe et al., 2008). Finally, it was reported that the functions and pharmacology of extra-striatal A_2A_ receptors must also be taken into account (Shen et al., 2008), although their function could not be totally extrapolated from the available data for striatal A_2A_ receptors. In this sense, the apparent controversial data obtained from pharmacological studies and genetic approaches using KO animals (presented in **Supplementary Tables S1, S2**) must be carefully exposed as the genetic background effects in A_2A_ KO animals may invalidate them as a model to study A_2A_ receptors (Filip et al., 2012). To conclude, the exploitation of the full potential of AR as drug targets will not only necessitate a full comprehension of AR-mediated mechanisms, but will also require the availability of ligands which let us distinguish among the different receptor populations discussed in this paper (Popoli and Pepponi, 2012).

## Misuse of Legal Psychostimulants

The consumption of legal psychostimulants has increased over recent years. For example, the misuse of prescribed psychostimulants, which are approved for the treatment of attention deficit hyperactivity disorder, for weight control or for the treatment of narcolepsy (Phillips et al., 2014), both by the patients themselves, and by non-affected individuals, based on misconceptions or simple lack of knowledge of the associated risks, is becoming more and more common nowadays (Lakhan and Kirchgessner, 2012; McHugh et al., 2015). This has been the case for methylphenidate consumption among college students as a study aid to enhance their academic performance (Maier et al., 2013; Webb et al., 2013; Vrecko, 2015). Although their use as a study aid is not the only reason why these substances are consumed (Drazdowski, 2016), this is one historically significant example, with reports of the use of amphetamines as study aids dating back to 1937 (Strohl, 2011).

In addition, caffeine, which is the most consumed psychoactive drug in the world, could also be of particular importance when addressing psychostimulant or drug addiction-related problems. Indeed, caffeine is commonly found as an adulterant in the preparation of illicit drugs (Prieto et al., 2016) and in energy drinks consumed in combination with alcohol or other psychostimulants (Reissig et al., 2009; Vanattou-Saïfoudine et al., 2012; Ferré, 2016). Interestingly, both acute and chronic adverse effects rise following concurrent consumption of caffeine and psychostimulant drugs. Specifically, caffeine worsens the psychostimulant’s toxicity by increasing hyperthermia, cardiotoxicity, and seizures, as well as influencing the stimulatory, discriminative, and reinforcing effects of psychostimulant drugs. These effects have been investigated for the cases of MDMA and cocaine ingested with caffeine (Comer and Carroll, 1996; Kuzmin et al., 1999; Vanattou-Saïfoudine et al., 2012; Górska et al., 2017). The molecular mechanism underlying the action of caffeine is the antagonism of AR, with A_1_ and A_2A_ subtypes the most involved. Indeed, it has been reported that caffeine induces increased DA release through A_1_ receptor blockade (Okada et al., 1997). More recently, findings by Ferré (2016) attribute caffeine potentiation of the psychomotor activating and reinforcing effects of psychostimulants to the existence of A_2A_/D_2_ heteromers, where the antagonism of A_2A_ receptors by caffeine reverts the inhibitory brake exerted by adenosine on D_2_ receptor signaling. Therefore, as ingestion of caffeine with cocaine and MDMA can significantly alter the drug-induced effects, understanding the molecular mechanisms underpinning this interaction will help to define correct approaches for the management of these side effects and toxicity.

## Concluding Remarks and Future Prospects

Although the A_2A_ receptor has been far more extensively studied (refer to **Supplementary Table S1** for summary), some papers considered in this review do highlight the role of A_1_ receptor activation to modulate psychostimulant-mediated effects. The properties of A_1_ agonists, mainly CPA, as anxiolytics have been previously reported in mice lacking A_1_ receptors (Giménez-Llort et al., 2002), in animal models of cocaine or alcohol consumption (Prediger et al., 2006; Hobson et al., 2013; O’Neill et al., 2014), and in classical behavioral studies (Jain et al., 1995). In this sense, electrophysiological studies in basolateral amygdala reported that application of CPA inhibits excitatory postsynaptic currents and glutamate release (Rau et al., 2014). This evidence, and the experiments reported in this review, will make A_1_ receptor signaling an important target for the development of novel pharmacological treatments for the common anxiety-like disorders reported during the process of drug-seeking and withdrawal, and as such will produce lasting changes in relapse susceptibility.

Evidence for the role of A_2A_ receptors in psychostimulant-mediated effects seems to be somewhat contradictory between different pharmacological studies, experimental models tested (particularly self-administration vs. experimenter-administration) and genetic approaches using A_2A_ KO animals (refer to **Supplementary Table S2** for more detail). However, the overall analysis of the presented data indicates that excitatory modulation of GPCR heteroreceptor complexes, in this case A_2A_/D_2_ heteroreceptors using A_2A_ agonists, is a promising tool to counteract psychostimulant-induced effects. Indeed, a prominent role in the modulation of psychostimulant addiction attributed to adenosine is mediated through A_2A_ activation by complex mechanisms, affecting various aspects of this phenomenon including locomotor activity, discrimination, seeking behavior and reward.

In this review, we have discussed current scientific evidence mainly based on animal models of psychostimulant addiction, and suggested promising candidates in the search for pharmacological interventions. This is particularly important because, nowadays, the main treatments against psychostimulant addiction are focused on behavioral interventions (Phillips et al., 2014), while AR pharmacology could be a powerful weapon to modify the neurochemical alterations that occur during psychostimulant addiction. AR are widely distributed in the CNS where they mediate a myriad of functions and interact with other neurotransmitter systems, which provides an opportunity to modulate specific complex brain functions. In addition, selective ligands are available for the different AR subtypes, which increase the chances to achieve nuclei-specific modulation, representing a pharmacological opportunity to control addictive psychostimulant consumption and health-related problems. Certainly, identifying strategies to fully understand AR signaling in drug addiction may provide insight into the factors contributing to consumption/craving/relapse of abused psychostimulants, thus revealing novel therapeutic approaches. We suggest that efforts could be made in three main aspects of adenosine pharmacology affecting psychostimulant addiction. Firstly, we have stated in this review that there is broad experimental evidence that pharmacological stimulation of A_1_ and A_2A_ receptors may counteract the effects induced by psychostimulants of abuse, but it is also important to highlight that approaches including a combination of AR drugs, like A_1_/A_2A_ ligands, may help form a more robust strategy when AR are the basis of pharmacological interventions. Secondly, as stated in Section “Mechanisms of Adenosine Receptor-Mediated Pathways in Drug Addiction,” due to the ability of AR to form homomers, heteromers and oligomers, it is mandatory to obtain specific ligands capable of discriminating among those different receptor populations. Thirdly, due to the lack of information concerning the effects consequent to alcohol and psychostimulant co-abuse, which is very common in drug addiction (Althobaiti and Sari, 2016; Barrett et al., 2016; Sánchez-López et al., 2017), it would be of particular interest to investigate the role of AR in those interactions.

## Author Contributions

CC developed the original idea. SG and CC designed the review. IB-Y and SG prepared the images. SG and CC prepared the tables. CC and SM edited and reviewed the final version of the article. All listed authors contributed to writing the article.

## Conflict of Interest Statement

The authors declare that the research was conducted in the absence of any commercial or financial relationships that could be construed as a potential conflict of interest.

## Footnotes

1 ClinicalTrials.gov

## Supplementary Material

TABLE S1 The role of adenosine receptor (AR)’s ligands in psychostimulant-induced effects upon behavior and function. The most relevant animal studies on the interaction between adenosine and psychostimulants are shown. Arrows represent decrease (↓) and increase (↑). When adenosine ligands induced no effect, the symbol “≈” is used. Although PAP9704 and ginsenosides are not ligands of AR, the effects of those ligands are reported in this table as authors suggests that their biological effects are mediated by AR.

TABLE S2 Genetic manipulation of AR in murine models and psychostimulant-induced effects. Genetic models derived from the manipulation of AR used to explore the interaction between adenosine and psychostimulants are shown. Arrows represent decrease (↓) and increase (↑). The symbol “≈” is used to indicate no difference between the genetic model and the wild-type animal (^∗^Unless other comparison is stated).

TABLE S3 Most relevant human clinical studies targeting AR. Ordered by publication date, most relevant clinical studies carried out in humans targeting the relationship between AR and psychostimulant addiction are shown. For each reference it is described the type of study, the number of subjects enrolled and its main objective and results. The number of the clinical trial is provided for the studies registered at ClinicalTrials.gov.
